# Mpox after COVID-19 in Africa: Different epidemic, similar challenges

**DOI:** 10.4102/jphia.v16i1.874

**Published:** 2025-03-31

**Authors:** Gloria P. Gómez-Pérez, Shem O.O. Sam, Nicaise Ndembi, Tobias F. Rinke de Wit

**Affiliations:** 1 Department of Research, PharmAccess Foundation, Amsterdam, the Netherlands; 2 Amsterdam Institute for Global Health and Development, Amsterdam, the Netherlands; 3 Department of Statistics, Faculty of Mathematics, Great Lakes University, Kisumu, Kenya; 4 Africa Centres for Disease Control and Prevention (Africa CDC), Addis Ababa, Ethiopia; 5 Department of Global Health, Faculty of Medicine, University Amsterdam UMC, Amsterdam, the Netherlands

**Keywords:** COVID, mpoxoutbreak, public health response, epidemic preparedness, zoonotic transmission, healthcare resilience in Africa

## Abstract

Africa faces again a new outbreak of mpox, heavily burdening the Democratic Republic of Congo (DRC). Factors such as biological and ecological changes in the virus, waning of immunity to smallpox, socio-economic challenges, and global economic strain are fueling this epidemic. To analyse the drivers of the mpox outbreak in DRC, review the international and local response, and draw lessons from the COVID-19 pandemic to propose strategies for building epidemic-resilient healthcare systems in the region. The focus is on the DRC, where the mpox epidemic is concentrated, and the broader African region, assessing both rural and urban healthcare contexts. This study synthesises epidemiological data, global health policies, and local health system responses. Key insights are drawn from COVID-19 case studies, and assessment of access to diagnostics and vaccines. The DRC hosts over 95% of Africa’s mpox cases, with significant challenges in human-wildlife interactions, poverty, and weakened healthcare systems exacerbated by COVID-19. Vaccine shortages, diagnostic delays, and uneven international support reflect a repeat of challenges seen during COVID-19. Localised vaccine and diagnostics production, improved risk communication, and digital health tools are highlighted as critical interventions. An epidemic-resilient healthcare framework, leveraging local production of medical products, regulatory empowerment, and real-time data collection, is essential for controlling mpox and future outbreaks. This study underscores the need for African-led solutions, addressing socio-economic drivers, enhancing local capacities, and fostering international solidarity to mitigate future epidemic threats.

## Introduction

Mpox is a zoonotic infectious disease caused by the monkeypox virus (MPXV) of the *Orthopoxvirus* genus. Other viruses belonging to this genus are variola (smallpox) and cowpox. Although mpox first gained international attention during the 2022‒2023 outbreak, MPXV has been circulating for decades in several African countries, resulting in several thousand reported human cases.^1^ The suspected animal reservoir of mpox are small forest-dwelling rodents (mice, rats, squirrels)^1^ that get in contact with humans when hunted for consumption or trade in African rural areas (animals bite or scratch and raw meat consumption), or when illegally imported to non-endemic settings. Human-to-human transmission can also occur and requires skin-to-skin contact, prolonged close contact, or indirect contact with contaminated materials in the household, healthcare facilities or through sexual contact.^1,2^ Vertical transmission from mother-to-child via the placenta is also possible.^2,3^ The Congo Basin mpox clade I (Central Africa clade) causes more severe disease than the West Africa clade II. Cameroon is the only country in which both clades concurrently circulate in the eastern and western rainforests for clade I (close to Democratic Republic of Congo [DRC]) and clade II (close to Nigeria), respectively.^4^ Scientific reports from few years ago describe the reproduction number (R0) of clade I to be between 0.6 and 1.0, and of clade II R0 presume to be lower.^5^ Based on this, sustained human-to-human transmission of mpox has been considered highly unlikely and outbreaks were expected largely from spillover events from zoonotic hosts.^5^ However, in the meantime, MPXV mutated, and two new subclades have been identified, named Ib and IIb, both linked to human-to-human transmission, including sexual contact. Subclade IIb caused a large mpox outbreak 2 years ago affecting 116 non-endemic countries with more than 90 000 cases worldwide.^6^

This global epidemic followed the reemergence of clade II in Nigeria between 2017 and 2019, which happened after 32 years of no reported cases. Local researchers in 2020 identified possible changes in the ecology (moving to urban areas) and transmissibility of MPXV clade II in Nigeria.^5^ Overall, the recent mutations of both clades, accompanied by the waning of immunity of the general population to smallpox ‒ that confers circa 85% cross protection to mpox^7^ ‒ have created an immunological niche for the resurgence of MPXV in the African region.^8^

## Possible factors driving the reemergence of mpox in Democratic Republic of Congo

Democratic Republic of Congo carries the highest burden of mpox, with more than 95% of all reported cases in Africa.^6^ The current outbreak in DRC is caused by subclades Ia and Ib. Subclade Ia was identified in most mpox endemic provinces in DRC, possibly associated with zoonotic spillover, and has a case fatality rate (CFR) of around 5% in some provinces.^9^ Subclade Ib is affecting East-DRC cities in a previously non-endemic province, South-Kivu. It is associated with human-to-human transmission and sexual contact, with a CFR of 1%.^9^ In addition, a unique situation is taking place in Kinshasa where both subclades Ia and Ib are co-circulating.^10^ Evidence of the suspected zoonotic spillover of clade Ia comes from recent analysis showing genetically diverse MPXV lineages co-circulating during the same outbreak in small geographic areas, suggesting multiple zoonotic introductions over a short period of time.^6^ Another piece of evidence supporting continued animal-to-human transmission of clade Ia is that it shows a low number of mutations induced by interactions with the human immune system. However, clade Ib (and IIb) shows a larger number of these mutations,^6,11^ suggesting longer contact with humans and adaptations for human-to-human transmission. To control the spread of mpox in DRC in the long term, it is crucial to understand the driving factors of the reemergence of mpox in the country, especially since the introduction of new lineages of clade Ia into densely populated cities such as Kinshasa pose a threat for additional regional and international distribution of MPXV clade I.^6^ Herewith, the key driving factors are described.

### Poverty

Democratic Republic of Congo is the fourth poorest country in the world.^12^ About one out of six people living in extreme poverty in sub-Saharan Africa (SSA) lives in DRC.^13^ The country’s Human Capital Index is 0.37, below SSA’s average of 0.4, reflecting decades of conflict, fragility and restricted development. The main contributors to this low score are high mortality rates under age five, high child stunting (42% of children under age 5 years) and low quality of education. In DRC, malnutrition is the underlying cause of almost half of the deaths of children < 5 years. In contrast with other African countries, the prevalence of stunting in the DRC has not decreased over the past 20 years.^13^ Displacement of millions of internal refugees from conflict zones to crowded camps with poor living conditions contribute to this outcome.^14^ During the current mpox outbreak, 85% of mpox-associated deaths in DRC are children < 15 years,^1^ likely malnourished. Among them, infants < 12 months old with mpox have a mortality rate four times higher than adults. Hence, poverty and low nutritional status (linked to impaired immune response to infections)^15^ are important driving factors for the reemergence of mpox in DRC (and elsewhere in SSA).

### Human–wildlife interaction

The high prevalence of potentially zoonotic spillovers of clade Ia in DRC mpox endemic provinces is remarkable. Democratic Republic of Congo has vast forests. Two-thirds of the country is covered in forest, with over half of the country’s population living in such remote and rural areas, mostly relying on the Congo Bassin forests for food, fuel and income.^16^ Human impact on the environment and biodiversity loss is increasing, but it is difficult to break the cycle of poverty of these populations that need the forest for survival.^16^ The distribution of several mpox cases along the Congo river and its affluents, in relation with traffic, might imply that the animal reservoir is spreading to these zones too, increasing the distribution of mpox along the river to cities such as Kinshasa.^6^ Many questions remain unanswered regarding the ecological factors of this outbreak: is there an increase in the animal-human contact? is there an increased prevalence of the virus among the animal host? has the animal host population increased^6^ or spreading to new geographic areas?

Research conducted in other African countries revealed that wildlife hunting and consumption increase when alternative livelihoods collapse and represents a safety net for people living near harvestable wildlife.^17^ In addition, the trade of terrestrial wildlife is an important source of income for many low-income families in these settings. Hence, one can only wonder if the current economic crisis in DRC could have increased human–wildlife interactions with subsequent increase of zoonotic spillovers.

### Impact of the COVID-19 pandemic and the war in Ukraine

Coronavirus disease 2019 (COVID-19), soaring inflation and the war in Ukraine, which led to high food prices, worsened the bad economic situation in Africa.^12^ And this certainly holds for DRC, where these issues are exacerbated by prolonged internal conflicts and civil war leading to mass migrations.^13^ The COVID-19 pandemic has truly undermined the fragile health systems in Africa, which in combination with environmental destruction and climate change, suffer from repeated disease outbreaks such as cholera, measles, Ebola, and now mpox. The COVID-19 also had a negative impact on the utilisation of health services in DRC since March 2020 with a decrease in hospital visits, a reduction in the number of antenatal care visits, reduced access to family planning and contraception and increased food insecurity.^13^ It is highly possible that this temporary interruption of basic healthcare delivery contributed significantly to a secondary health crisis in DRC, explaining the higher CFRs of clade Ia mpox cases in poorer provinces like Équateur.^9^

## Lessons learnt from the COVID-19 pandemic

During COVID-19, most industrialised countries adopted a narrow and nationalistic view about the pandemic; with a blind spot for poor nations when it came to vaccine and medical aid distribution. A shift at governmental and pharmaceutical industry levels is needed to avoid ‘stockpiling’ medicines and vaccines. Even now less than 40% of the African population has received a full dose of the COVID-19 vaccine,^18^ while other nations have more than 80% coverage, in addition to several booster doses. Hence, it seems like in times of global health emergencies, an emphatic and solidary attitude towards countries with very limited economic resources is a goal that is too optimistic. The alternative, namely the more common-place self-interest argument would probably be better. Self-interest of developed countries would justify ring-fencing vaccination strategies in Africa to contain epidemics in their area of origin, instead of waiting for those to become global. It took COVID-19 omicron variant 2 weeks to move from South Africa to Europe.

### Create local markets for vaccines in Africa

Taking the above-stated into account, the advance purchase agreement (APA) was created after the COVID-19 pandemic to ensure rapid access to scarce vaccine supplies in future health emergencies.^19^ The APA is funded by Gavi’s First Response Fund and was activated as soon as the World Health Organization (WHO) declared mpox a Public Health Emergency of International Concern (PHEIC) on 14 August 2024. Although at the moment of this submission, around 6.1 million mpox vaccines have been donated according to Africa Centres for Disease Control and Prevention (Africa CDC) mpox vaccine donation tracker, shipping 250 000 mpox vaccines from Europe to DRC around 1 month after the PHEIC declaration, entailed passing quality control and other regulatory requirements that delayed the availability of the vaccines on-site in Africa. The longer-term solution to such problems is creating vaccine manufacturing capacity in Africa supported by regional regulatory arrangements under Africa CDC. Initiatives such as Partnerships for African Vaccine Manufacturing (PAVM)^20^ need to become a reality, a path Gavi is willing to support through the African Vaccine Manufacturing Accelerator (AVMA).^21,22^ In addition, economic endorsement by international donors and organisations like the WHO, Gavi and United Nations Children’s Fund (UNICEF) needs to be increasingly paired by local government commitment in Africa.^23^ African leaders could create new business models for pharmaceutical companies where the expected profit can be realistically tuned to the economic capacity of poor nations.

### African production of pharmaceutical products, medical supplies and diagnostic tests

Africa imports 99% of its routine vaccine needs and 95% of its medicines.^24^ It accounts for 25% of the global demand, but less than 1% of the global supply.^18,25^ It is estimated that Africa’s vaccine needs is of around 1.5 billion doses annually, that can surpass 2.7 billion by 2040.^23^ Hence, there is urgency to move initiatives like PAVM forward; maybe starting in the countries with some pharma manufacturing capacity like South Africa, Senegal, Morrocco and Egypt.

Likewise, diagnostic tests need to be produced locally in Africa. For instance, there is a disturbing contrast between where the mpox outbreak occurs (all African countries) and where the mpox diagnostic tests are produced (all non-African countries).^26^ An international step forward is the 100 Days Coalition, which aims to prioritise diagnostics, therapeutics and vaccines with broad coverage and little need for updating for the top 10 pathogens of pandemic potential.^27^ An inclusive partnership within this coalition, co-designing products with affected communities,^27^ would need an active participation of Africa. Gavi has also launched in June 2024 the AVMA. This financing instrument will make up to $1.2 billion available over 10 years to support the sustainable growth of Africa’s manufacturing base.^21^ These initiatives take shape in collaborative efforts with local agencies and partnerships like PAVM.

### Empower African medicine regulatory agencies

In addition, recommendations from large international regulatory agencies like the United States (US) Food and Drug Administration (FDA), or the WHO and the European Medical Agency (EMA) in Europe are developed for the use of pharmaceutical products in the Global North. If followed in Africa, their recommendations can represent an impediment rather than a facilitator of swift licensing, production and distribution of medicines, vaccines or diagnostic tests. The African Medicines Agency (AMA) should carry this responsibility, with Africa CDC and the African Union Development Agency-New Partnership for Africa’s Development (AUDA-NEPAD) being in the key position to guide expedited regulatory procedures on a continental basis, to be implemented by local regulatory agencies in African countries. Positive developments towards this direction are the recent recommendations (23 September 2024) of Africa CDC Diagnostic Advisory Committee on appropriate high-quality molecular tests to procure and use for the mpox response in Africa. The recommendation includes a list of nine mpox molecular diagnostic tests with manufacturers in the US, Germany, Spain, China and South Korea.^28^

### Risk communication and community engagement

A lack of official communications and misinformation in low- and middle-income countries (and worldwide) were big problems during the COVID-19 pandemic. In DRC, there has been significant COVID-19 vaccine hesitancy, with limited demand.^13^ Studies in SSA showed that willingness to get vaccinated is discouraged by a lack of easy access to vaccines at the local level.^29^ In addition, social ties and perceptions, religion, cultural narratives and intra-household power relations influences vaccine take-up.^29,30^ This can happen also when not having access to the Internet. Therefore, it is critical for a successful public health intervention to develop culturally sensitive public health communication strategies to address the fears and beliefs of the population.^30^ For instance, credible radio broadcasts (that have widespread reach) and medical professionals are highly trusted.^29^ Risk communication and community engagement (RCCE) strategies should be an inherent component of the response to epidemics^31^ and recommended by the WHO.

### Support real-time African data collection

In the absence of prompt epidemiological data on COVID-19, Africa public health strategies basically followed guidelines developed in developed countries, including curfews, social distancing, prolonged closure of schools; measures that are not realistic and, on top of that, disproportionally detrimental to fragile African economies. Also, recommendation of mass vaccination against COVID-19 in industrialised settings when applied in Africa, precluded targeted interventions in the continent, which would have been much more realistic based on local demographics and epidemiology of COVID-19. The average age of an African is over two decades younger than in Europe with concomitant lower COVID-19 symptomatology. In addition, Africans’ immune systems are generally more activated than Europeans, resulting in less COVID-19 severe cases.^32^ Therefore, vaccines would have been much more impactful when specifically administered to vulnerable population groups. However, once the (limited) vaccines arrived on the continent, African nations had no clarity about which populations to prioritise (demographically, socio-economically) and where these populations resided geographically.

## A case study of swift local data collection and translation into policy

**FIGURE 1:**
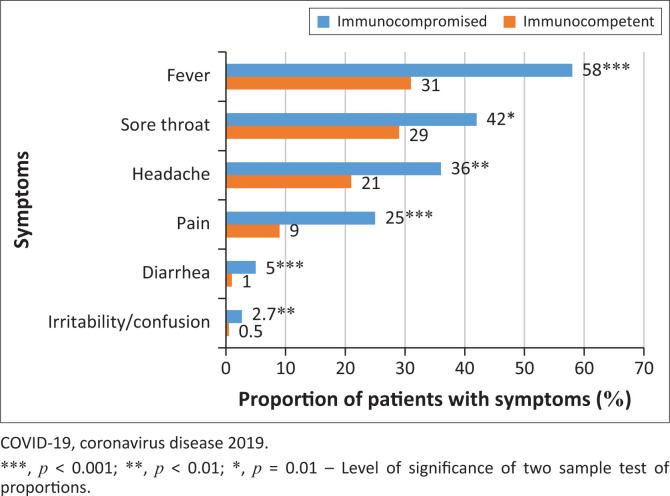
Proportion of COVID-19 positive immunocompromised and immunocompetent patients presenting with indicated symptoms.

Here, we share our experience with a digital COVID-19 surveillance project (‘COVID-Dx’) developed in close collaboration with a West Kenyan consortium, the Lake Region Economic Bloc (LREB). Over less than a month, we deployed a digital data entry system in KoboToolbox to capture COVID-19 cases and identify vulnerable populations in an area that covers 15 million Kenyans. Data analysis of COVID-19 health services from 111 healthcare centres digitally connected within 14 Counties in LREB from April 2021 to December 2022 revealed important results. Our focus here is on the effects of COVID-19 in immunocompromised patients. Among the 62 188 patients with known COVID-19 status (polymerase chain reaction [PCR] or/and rapid-test results) in our system, 9036 patients tested COVID-19 positive, of whom 213 were immunocompromised. We found that the proportion of immunocompromised patients among COVID-19 positive individuals was significantly higher than among the COVID-19 negative (0.81% vs. 0.26%; *p* < 0.001). Also, the risk of being diagnosed with COVID-19 was three times higher in immunocompromised compared with immunocompetent patients (*p* < 0.001). Among COVID-19 positive patients, the immunocompromised had 2‒5 times more frequent COVID-19 symptoms such as fever, sore throat, headache, pain, diarrhoea and irritability and/or confusion (Figure 1). Moreover, they were 2.5 times more likely to be hospitalised compared to COVID-19 positive immunocompetent individuals (*p* = 0.01). Fully vaccinated immunocompromised patients with COVID-19 were 91% less likely to be hospitalised than unvaccinated immunocompromised COVID-19 positive patients (*p* = 0.01). Importantly, we also found that a single dose of the vaccine did not significantly protect immunocompromised individuals from hospitalisation. Other research on people living with HIV shows that they might benefit from a third dose of the COVID-19 vaccine.^33^ People living with HIV who are untreated or uncontrolled are also at higher risk of severe mpox and related death.^34^

**FIGURE 2:**
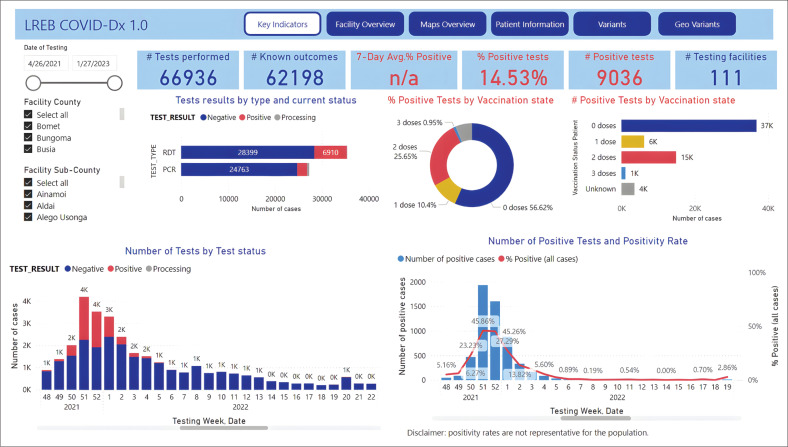
Screenshot of Power-BI COVID-Dx dashboard front page, Lake Region Economic Bloc depicting the 2021–2022 Omicron wave.

**FIGURE 3:**
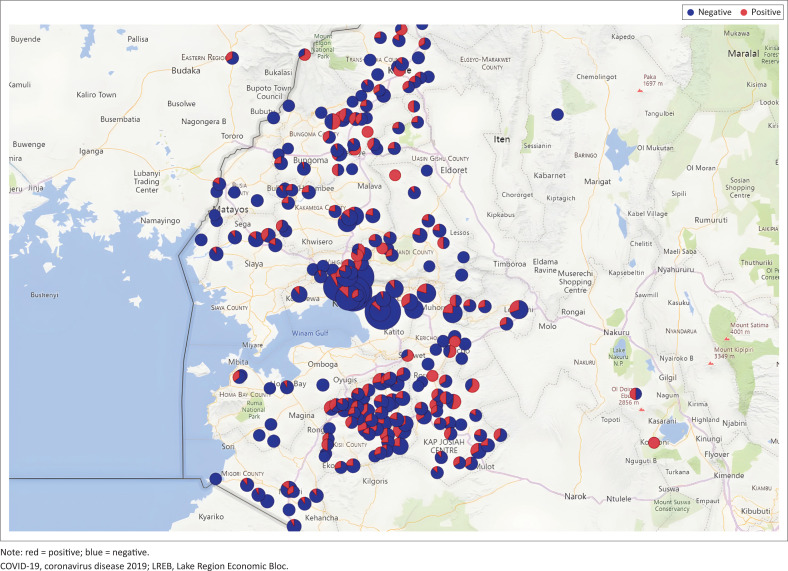
Cumulative geo-information on COVID-19 Omicron incidence LREB, West Kenya.

During this project, data were captured in mobile phone dashboards in semi-real-time and accessible to all participating facilities, as well as to and local health authorities up to the Ministries of Health of 14 counties in West Kenya (Figure 2 and Figure 3). The results of these analyses were shared immediately with pertinent local health authorities and mainstream media. This resulted in several Policy Briefs^35^ and LREB Advisories that were promptly developed and supported action almost in real-time. Thus, it was demonstrated that in times of crisis, digital health data collection and mobile phone-based analytics, including time-tagged and geo-mapped data, can be collected in the African setting and support concomitant policy decisions. This level of timeliness and precision was not even reached in most European countries.

Unfortunately, when at the end of the epidemic the project was transferred to LREB, and with the declining COVID-19 cases, these intensive surveillance efforts discontinued. This highlights that ad hoc funding is not sufficient to increase epidemic surveillance systems, but that prolonged local investments are required in human resources and technological capacity, as well as a more flexible approach that can hibernate in times of low epidemiological pressure.

All in all, digital tools can be a great asset to assess and improve epidemic preparedness in Africa, helping reach centres in remote locations and to decentralise epidemic control efforts. Such tools need to be adapted to the African context, for example, taking into consideration limits in network connectivity, data band width and data server hosting. For instance, this can be addressed by developing tools that allow uploading data without Internet connection.^36,37^

## Moving forward

Democratic Republic of Congo has a national surveillance system in place since 2001.^6^ This has allowed to follow the unfolding of the current outbreak throughout the country. Nevertheless, ongoing conflicts hinder access to affected areas and the response efforts. Even in the presence of a robust surveillance system, remote health facilities in DRC are not sufficiently equipped to safely collect skin lesion swab samples, and instead blood samples are collected and sent to reference laboratories.^6^ Hence, local researchers and health providers call for action to provide facilities with appropriate materials to improve the molecular diagnosis of MPXV and decentralise testing. When diagnostics tests are lacking, there is uncertainty about the real situation regarding the epidemiology of ongoing outbreaks.^6^ In remote areas, there is less availability of trained personnel. Therefore, in Africa there is a great need of alternative diagnostic tools that do not require expensive equipment and highly skilled laboratory workers. In this sense, we are of the view that *the perfect (preferred) diagnostic tests should not be the enemy of the good*. According to the WHO the preferred diagnostic test for mpox is molecular diagnosis by PCR. But even in reference laboratories in DRC, cartridges for GeneXpert machines are currently scarce.^9^ New, easy to perform and affordable mpox tests are recommended by Africa CDC.^28^

Evidently, the mpox outbreak in DRC has been neglected for many years and today we are paying the price for this non-epidemic preparedness. All the same, there are some positive changes that took place after the tough experience of the COVID-19 pandemic. The call for mpox vaccine donations could indeed have happened earlier, but it has generated relatively rapid positive results: 6.1 million vaccines have been donated, equivalent to approximately two-thirds of total needed. With this shortage, targeted prioritisation of vaccine allocation in Africa is needed guided by best possible real-time knowledge of the populations at highest risk. The parallel urgency of local manufacture of vaccines, medicine supplies and diagnostic test should be addressed too and not wait for the next public health emergency. We have the knowledge and tools to control this epidemic and should deploy those, whether by solidarity, humanity or self-interest. Like with the eradication campaigns of smallpox, a highly coordinated global effort and commitment at all levels is needed to keep mpox under control, and perhaps eradicating MPXV too. This commitment requires creating significant independence from external resources in Africa by creating local markets, producing local medical supplies and utensils, empowering local regulatory mechanisms and generating real-time local data. Mpox after COVID-19 in Africa is a different epidemic, with similar challenges. Disease X after mpox will be different again, but its challenges are predictable and actionable for the continent.
